# Hypernatremia in Hospital-at-Home Patients: Prevalence, Clinical Profile, and Mortality in Institutionalized and Home-Dwelling Older Adults

**DOI:** 10.3390/medsci14020206

**Published:** 2026-04-22

**Authors:** María de Castro-García, Sara Núñez-Palomares, Juan Miguel Antón-Santos, Alejandro Estrada-Santiago, Yolanda Majo-Carbajo, Pilar García de la Torre-Rivera, Francisco Javier García-Sánchez, Pilar Cubo-Romano

**Affiliations:** 1 Hospital-at-Home Unit, Internal Medicine Service, Hospital Universitario Infanta Cristina, Instituto de Investigación Sanitaria Hospital Puerta de Hierro Segovia Arana (IDIPHISA), 28981 Madrid, Spain; mcastrog@salud.madrid.org (M.d.C.-G.); snpalomares@salud.madrid.org (S.N.-P.); jmanton.hugf@salud.madrid.org (J.M.A.-S.); alejandro.estrada@salud.madrid.org (A.E.-S.); yolanda.majo@salud.madrid.org (Y.M.-C.); mpilar.garciade@salud.madrid.org (P.G.d.l.T.-R.); pilar.cubor@salud.madrid.org (P.C.-R.); 2 Emergency Room Service, Surgical Prehabilitation Unit, Hospital Universitario Infanta Cristina, Instituto de Investigación Sanitaria Hospital Puerta de Hierro Segovia Arana (IDIPHISA), 28981 Madrid, Spain; 3 Department of Medicine, Faculty of Medicine, University Complutense of Madrid, 28040 Madrid, Spain

**Keywords:** hypernatremia, Hospital-at-Home, frailty, older adults, institutionalized patients, mortality

## Abstract

Background: Hypernatremia is an infrequent but clinically relevant electrolyte disorder in older adults and is associated with poor outcomes. Patients managed through Hospital-at-Home (HaH) programs, particularly those living in institutional settings, are especially vulnerable due to functional dependency and cognitive impairment. Evidence regarding the prevalence and prognostic impact of hypernatremia in HaH settings remains limited. Methods: We conducted a retrospective observational cohort study including all patients admitted to a Hospital-at-Home unit between 2019 and 2024. Patients were classified according to care setting as home-dwelling or institutionalized. Hypernatremia was defined as a serum sodium concentration >145 mmol/L. Sociodemographic, functional (Barthel Index), and cognitive (Global Deterioration Scale) variables were collected. Mortality during HaH admission and at 30, 60, and 90 days was analyzed, and survival was assessed using Kaplan–Meier methods. Results: A total of 4501 patients were included, of whom 2701 were treated at home and 1800 in institutional settings. Hypernatremia was significantly more prevalent among institutionalized patients than among home-dwelling patients (3.1% vs. 0.8%, *p* < 0.001). Institutionalized patients with hypernatremia showed greater functional dependency (Barthel Index 11 vs. 15, *p* = 0.041) and more advanced cognitive impairment (GDS 6 vs. 5.5, *p* = 0.033) compared with those without hypernatremia. Mortality among institutionalized patients with hypernatremia was high, reaching 32.9% during HaH admission, 61.2% at 30 days, 70.6% at 60 days, and approximately 79% at 90 days. Kaplan–Meier analysis demonstrated a rapid decline in survival during the first month following diagnosis. Conclusions: In Hospital-at-Home programs, hypernatremia is more prevalent among institutionalized older adults and is strongly associated with severe functional and cognitive impairment and very high short- and medium-term mortality. These findings suggest that hypernatremia should be considered a marker of advanced frailty rather than an isolated electrolyte disturbance.

## 1. Introduction

Hypernatremia is an electrolyte disorder characterized by an elevation of serum sodium concentration above 145 mmol/L, reflecting a state of relative water deficit. Although relatively uncommon in the general population, hypernatremia is disproportionately observed among older adults and is consistently associated with adverse clinical outcomes, including prolonged hospitalization, functional decline, and increased mortality. In geriatric patients, hypernatremia often develops in the context of frailty, cognitive impairment, functional dependency, and a reduced ability to maintain adequate oral fluid intake [1,2].

Previous studies have shown that hypernatremia in older adults is frequently not an isolated biochemical abnormality but rather a clinical marker of severe vulnerability. Factors such as impaired thirst perception, dysphagia, dependency for basic activities of daily living, and polypharmacy contribute to its development and persistence. Several hospital-based cohorts have reported markedly increased short- and medium-term mortality among elderly patients with hypernatremia, suggesting that this condition may reflect advanced physiological reserve depletion rather than a reversible electrolyte imbalance alone [3,4,5].

Hospital-at-Home (HaH) programs have expanded substantially in recent years as an alternative to conventional hospitalization, particularly for older patients with complex chronic conditions. These programs aim to provide hospital-level care in the patient’s usual environment, either at home or in long-term care facilities, while reducing hospital-related complications. However, patients managed through HaH are often highly dependent, cognitively impaired, and clinically fragile, placing them at increased risk of hydration-related disorders. Despite this, data on the epidemiology and clinical impact of hypernatremia in HaH settings remain scarce [6].

Moreover, important differences may exist between patients receiving HaH care in their own homes and those institutionalized in nursing homes or long-term care facilities. Institutionalized patients frequently present with more advanced functional and cognitive impairment, higher levels of dependency, and limited autonomy in fluid intake, which may further increase the risk of hypernatremia and associated mortality. Understanding these differences is essential to tailor preventive strategies and optimize care models in both settings [7].

In addition to its classical pathophysiological interpretation as a water deficit state, hypernatremia has increasingly been recognized as a clinical marker of vulnerability in older adults. In this population, it rarely occurs as an isolated electrolyte imbalance but rather reflects a complex interplay of physiological decline, impaired homeostatic mechanisms, and dependency-related factors. The prevalence of hypernatremia increases significantly with age, particularly among institutionalized patients, where rates may exceed 10% in certain high-risk cohorts.

Older adults are especially susceptible due to age-related changes such as reduced thirst sensation, impaired renal concentrating ability, and decreased total body water. These physiological changes are compounded by clinical conditions including dementia, immobility, dysphagia, and polypharmacy, all of which contribute to inadequate fluid intake and increased risk of dehydration.

Despite growing recognition of hypernatremia as a prognostic marker, most evidence derives from conventional hospital settings, intensive care units, or emergency departments. In contrast, Hospital-at-Home (HaH) programs represent a unique and expanding model of care, characterized by the management of complex, frail patients outside traditional hospital environments. However, the epidemiology and prognostic implications of electrolyte disorders such as hypernatremia in HaH remain poorly understood.

Furthermore, the heterogeneity within HaH populations—particularly between patients treated at home and those in institutional settings—has not been sufficiently explored. Institutionalized individuals often exhibit higher levels of dependency and cognitive impairment, potentially modifying both the risk and clinical significance of hypernatremia.

Understanding hypernatremia in this context is essential not only from a clinical perspective but also for healthcare organizations, as it may serve as an early indicator of deterioration, unmet care needs, or advanced frailty requiring a shift in management strategies.

Therefore, the aim of this study was to evaluate the prevalence, clinical characteristics, and short- and medium-term mortality associated with hypernatremia in a large cohort of patients managed through a Hospital-at-Home unit. We specifically compared patients treated at home with those treated in institutional settings, hypothesizing that hypernatremia would be more prevalent and associated with worse outcomes among institutionalized patients, reflecting a higher degree of underlying frailty [8].

## 2. Materials and Methods

### 2.1. Study Design

We conducted a retrospective observational cohort study including all patients admitted to the Hospital-at-Home (HaH) unit of Hospital Universitario Infanta Cristina between January 2019 and December 2024. The study was designed to evaluate the prevalence and clinical impact of hypernatremia in older adults receiving hospital-level care in non-conventional settings.

### 2.2. Setting and Participants

The Hospital-at-Home unit provides acute and subacute hospital care to patients either in their private homes or in long-term care facilities, including nursing homes and residential institutions. Eligible patients were classified into two cohorts according to the care setting:

- Home-dwelling HaH patients (HaH-home)
- Institutionalized HaH patients (HaH-institution)

All consecutive patients admitted during the study period were included. No additional exclusion criteria were applied, ensuring a real-world representation of HaH activity.

### 2.3. Variables and Definitions

Sociodemographic variables included age and sex. Functional status was assessed using the Barthel Index, with lower scores indicating greater functional dependency. Cognitive status was evaluated using the Global Deterioration Scale (GDS). Polypharmacy was defined as the regular use of five or more medications.

Hypernatremia was defined as a serum sodium concentration >145.00 mmol/L at any point during HaH admission and was further classified as mild, severe, or extreme according to established thresholds. Clinical complications frequently associated with frailty were recorded, including dysphagia, aspiration events, and pressure ulcers [9,10].

### 2.4. Outcomes

The primary outcome was all-cause mortality during HaH admission and at 30, 60, and 90 days after the diagnosis of hypernatremia. Secondary outcomes included the prevalence of hypernatremia in each care setting and the association between hypernatremia and functional, cognitive, and clinical characteristics.

### 2.5. Statistical Analysis

Continuous variables are presented as mean ± standard deviation or median and interquartile range, as appropriate. Categorical variables are expressed as absolute numbers and percentages. Comparisons between groups were performed using Student’s *t*-test or Mann–Whitney U test for continuous variables and $\chi^{2}$ test for categorical variables.

Survival analysis was performed using Kaplan–Meier curves, with differences assessed by the log-rank test. Multivariable proportional hazards regression models were constructed to evaluate the association between hypernatremia and mortality, adjusting for age, sex, functional status (Barthel Index), cognitive impairment (GDS), and care setting (home vs. institutional). Results are presented as hazard ratios (HRs) with 95% confidence intervals (95% CI). A two-sided *p* value < 0.05 was considered statistically significant. Statistical analyzes were performed using standard statistical software [11].

Given the real-world nature of Hospital-at-Home care, a retrospective observational design was considered appropriate to capture the full spectrum of patient characteristics and clinical outcomes without introducing selection bias.

Data were extracted from electronic medical records and cross-checked to ensure consistency. In cases of missing data, complete-case analysis was performed, as the missingness was considered random and represented a small proportion of the dataset.

All statistical analyses were conducted using R studio version 2026.01.1+403, ensuring reproducibility and adherence to standard reporting practices. The selection of variables included in the multivariable model was based on clinical relevance and the previous literature.

Given the real-world nature of Hospital-at-Home care, a retrospective observational design was considered appropriate to capture routine clinical practice without introducing selection bias.

Missing data were minimal and handled using a complete-case analysis approach, assuming missingness at random. Variables included in the multivariable proportional hazards model were selected based on clinical relevance and the prior literature, including age, sex, functional status (Barthel Index), cognitive impairment (GDS), and care setting.

The proportional hazards assumption was assessed using graphical methods (log-minus-log survival plots) and was considered satisfied. Sensitivity analyses were performed by stratifying the sample according to care setting and severity of hypernatremia, confirming the robustness of the primary results.

### 2.6. Ethical Considerations

The study was approved by the local Research Ethics Committee (Code: PI 05/24 of 8 January 2024) of Hospital Puerta de Hierro—Majadahonda and conducted in accordance with the Declaration of Helsinki. Due to the retrospective nature of the study, informed consent was waived.

## 3. Results

### 3.1. Study Population

A total of 4.501 patients were admitted to the Hospital-at-Home unit during the study period. Of these, 2.701 (60.0%) were treated in their own homes (HaH-home), while 1.800 (40.0%) received care in institutional settings (HaH-institution). Baseline characteristics by care setting are shown in Table 1.

Institutionalized patients were significantly older than home-dwelling patients (90.10 ± 8.00 vs. 69.50 ± 18.0 years) and presented with markedly higher levels of functional dependency and cognitive impairment, as reflected by lower Barthel Index scores and higher Global Deterioration Scale stages.

### 3.2. Prevalence of Hypernatremia

**Figure 1:**
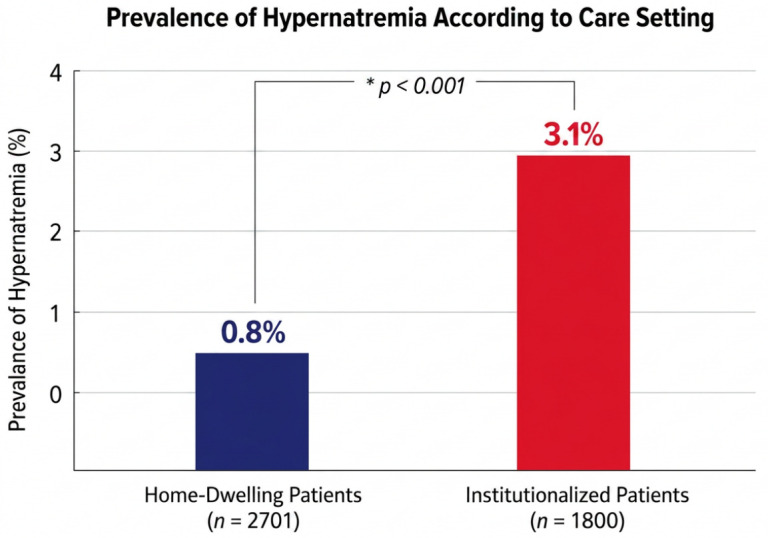
Prevalence of hypernatremia according to care setting in the Hospital-at-Home cohort. Hypernatremia was significantly more prevalent among institutionalized patients (3.1%) than home-dwelling patients (0.8%; *p* < 0.001; HaH-home: n = 2701; HaH-institution: n = 1800).

The overall prevalence of hypernatremia differed significantly between care settings. Hypernatremia was identified in 0.80% of patients treated at home and 3.10% of institutionalized patients (*p* < 0.001), resulting in a total of 84 patients with hypernatremia in the overall cohort (Figure 1).

Among home-dwelling patients, those who developed hypernatremia were older and significantly more functionally dependent than non-hypernatremic patients (82 ± 14 vs. 69 ± 18 years; Barthel Index 39 ± 37 vs. 83 ± 27; both *p* < 0.001).

Notably, the prevalence of hypernatremia in institutionalized patients was nearly four times higher than in home-dwelling individuals, suggesting a strong association with care dependency and environmental factors.

In contrast, among institutionalized patients, age did not significantly differ between those with and without hypernatremia (88.4 ± 8.2 vs. 90.4 ± 7.9 years). However, hypernatremic institutionalized patients showed significantly greater functional dependency, more advanced cognitive impairment, and a higher proportion of women compared to their non-hypernatremic counterparts (Table 1).

### 3.3. Clinical Characteristics of Institutionalized Patients with Hypernatremia

A total of 84 patients with hypernatremia were identified in the overall cohort. Among them, 57 corresponded to institutionalized patients, who were analyzed separately. The severity distribution (mild, severe, and extreme hypernatremia) was assessed in the full hypernatremic cohort (n = 84). Mean serum sodium concentration was 160.00 mmol/L (range: 146.00–189.00), and 32.00% of patients presented with extreme hypernatremia (>165 mmol/L). This approach allowed us to distinguish between overall severity patterns and subgroup-specific outcomes in institutionalized patients. (Table 2)

This subgroup exhibited severe frailty, with a mean Barthel Index of 11.6 and advanced cognitive impairment (mean GDS 5.8). Polypharmacy was common, with a median of six medications per patient. Frequent clinical complications included dysphagia (43.5%), aspiration events (32.9%), and pressure ulcers (30.6%) (Table 3). Among institutionalized patients, hypernatremia was consistently associated with markers of advanced frailty, including lower functional scores and higher cognitive impairment, reinforcing its role as a surrogate indicator of clinical vulnerability.

These findings reinforce the interpretation of hypernatremia as a marker of advanced frailty rather than an isolated biochemical alteration.

### 3.4. Mortality and Survival Analysis

Hypernatremia was associated with increased mortality (HR 1.85; 95% CI 1.30–2.65; *p* < 0.001). In-hospital mortality during HaH admission was 32.90%. Cumulative mortality increased to 61.20% at 30 days, 70.60% at 60 days, and approximately 79% at 90 days.

Survival analysis was specifically performed in institutionalized patients with hypernatremia (n = 57), given their higher clinical vulnerability.

**Figure 2:**
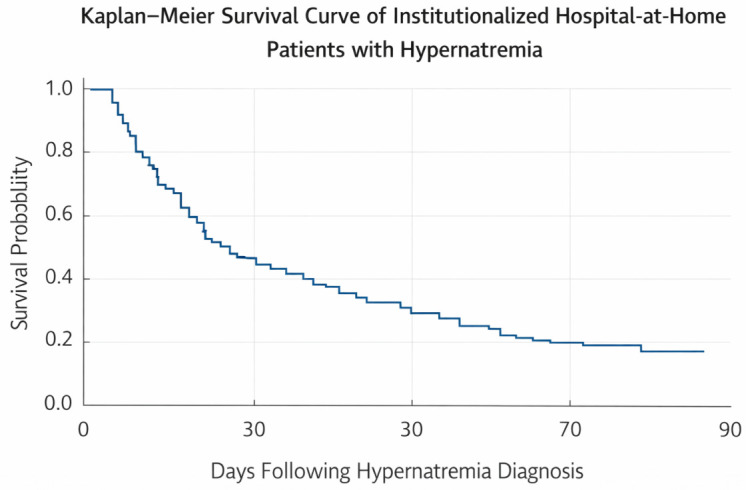
Kaplan–Meier survival curve of institutionalized Hospital-at-Home patients with hypernatremia (n = 57). Survival probability declines rapidly, reaching approximately 68% at admission end, 39% at 30 days, 29% at 60 days, and 21% at 90 days following hypernatremia diagnosis.

Kaplan–Meier survival analysis demonstrated a rapid decline in survival during the first month following hypernatremia diagnosis, with a trend toward poorer outcomes among patients with higher sodium levels (Figure 2).

In multivariable analysis adjusted for age, sex, functional dependency, cognitive impairment, and care setting, hypernatremia remained strongly associated with increased mortality (Table 4).

## 4. Discussion

Importantly, the distinction between the overall hypernatremic cohort and the institutionalized subgroup allowed for a more precise interpretation of severity and mortality patterns.

In this large real-world cohort of patients managed through a Hospital-at-Home (HaH) program, hypernatremia was an infrequent but clinically significant finding, particularly among institutionalized older adults. Our results demonstrate that hypernatremia was significantly more prevalent in institutional settings than among patients treated at home (3.1% vs. 0.8%) and was associated with profound functional dependency, advanced cognitive impairment, and extremely high short- and medium-term mortality. These prevalence figures align with prior reports from hospital settings, where hypernatremia affects 1–3% of older patients, rising to 10–12% among those admitted from nursing homes. Our findings support the conceptual shift from viewing hypernatremia as a purely biochemical abnormality to understanding it as a clinical syndrome associated with frailty and care dependency. In this sense, hypernatremia may represent the final pathway of multiple interacting factors, including functional decline, cognitive impairment, and insufficient caregiving support [12,13,14,15].

Our findings support a conceptual shift from viewing hypernatremia as a purely biochemical abnormality to understanding it as a clinical marker of vulnerability and advanced frailty in older adults.

These findings are consistent with previous hospital-based studies reporting hypernatremia as a strong predictor of adverse outcomes in older adults. For instance, large cohorts have documented mortality rates of 30–45% in hypernatremic elderly patients during hospitalization, escalating to over 70% in ICU or sepsis contexts, comparable to our 79% 90-day rate in institutionalized HaH patients. However, our study extends existing knowledge by specifically focusing on the HaH setting, an increasingly important model of care for frail elderly patients. In this context, hypernatremia appears less as an isolated electrolyte disturbance and more as a clinical marker of advanced vulnerability and physiological reserve depletion [3,8,11,12,14].

The comparison between home-dwelling and institutionalized patients provides important insights. While age and dependency were both associated with hypernatremia in home-dwelling patients, age alone did not discriminate risk among institutionalized patients. Instead, functional dependency and cognitive impairment emerged as the main distinguishing factors. This pattern mirrors data from care home cohorts, where nursing home residents showed a 10-fold higher hypernatremia risk (12% vs. 1.3% in own-home patients), largely attributable to dementia and dependency, even after multivariable adjustment. This observation supports the concept that biological frailty and neurocognitive dysfunction, rather than chronological age, are key determinants of hydration-related disorders in older adults. From a healthcare system perspective, these results highlight the need to integrate systematic hydration monitoring protocols within HaH programs. Institutionalized settings, in particular, may benefit from structured interventions, including fluid intake tracking, caregiver education, and early warning systems [11,12,13,16].

Although data specifically from Hospital-at-Home settings remain limited, studies conducted in long-term care and community-dwelling populations have reported similar associations between hypernatremia, dependency, and mortality, supporting the external validity of our findings [7,17].

Mortality among institutionalized patients with hypernatremia was strikingly high, approaching 80% at three months. The Kaplan–Meier survival analysis revealed a rapid decline in survival during the first month following diagnosis, highlighting the particularly poor short-term prognosis. Importantly, the identification of hypernatremia in highly dependent patients should prompt a reassessment of therapeutic goals. In many cases, it may reflect advanced disease stages where a palliative approach, focused on comfort and quality of life, may be more appropriate than aggressive correction strategies. Importantly, mortality did not differ significantly according to the degree of hypernatremia severity, suggesting that even moderate elevations in serum sodium may reflect a critical underlying clinical state. This is consistent with geriatric literature viewing hypernatremia not as a direct cause of death but as an indicator of inadequate care, severe frailty, or depleted physiological reserves in institutionalized settings. This reinforces the interpretation of hypernatremia as a marker of advanced frailty rather than a direct causal factor of death [3,7,11,12,14].

From a clinical and organizational perspective, these findings have relevant implications for HaH programs and long-term care facilities. Institutionalized patients often depend entirely on caregivers for fluid intake and basic needs, and subtle reductions in hydration may go unnoticed until severe biochemical disturbances develop. Studies in care homes link hypernatremia to preventable dehydration, with population-attributable fractions up to 36% for hospital admissions and 85% for in-hospital deaths. Early identification of patients at risk, systematic monitoring of fluid intake, and proactive hydration strategies may help prevent the development of hypernatremia or allow earlier intervention. Furthermore, prompt correction protocols are essential, as delayed or overly rapid management exacerbates outcomes. Furthermore, in patients with advanced dependency and cognitive impairment, the detection of hypernatremia should prompt a comprehensive reassessment of goals of care, including the timely integration of palliative approaches when appropriate [12,13,15,16,17].

From a healthcare system perspective, these findings highlight the need to implement structured hydration monitoring protocols within Hospital-at-Home programs, particularly in institutional settings.

Importantly, the identification of hypernatremia in highly dependent patients should prompt a reassessment of goals of care, including the consideration of palliative approaches when appropriate.

To our knowledge, this study represents one of the largest analyses of hypernatremia specifically conducted in a Hospital-at-Home setting.

## 5. Limitations

Several limitations of this study should be acknowledged. First, its retrospective observational design precludes the establishment of causal relationships between hypernatremia and mortality. Second, the study was conducted in a single HaH unit, which may limit generalizability to other healthcare systems with different organizational structures or patient profiles. Third, detailed data on fluid intake, thirst perception, and caregiver-related factors were not available, preventing a more granular analysis of the mechanisms leading to hypernatremia. Finally, although functional and cognitive status were systematically assessed, other potential confounders, such as comorbidity burden or inflammatory markers, were not included in multivariable analyses.

Additionally, the retrospective design precludes causal inference between hypernatremia and mortality. Therefore, hypernatremia should be interpreted as a marker of underlying frailty rather than a direct causal factor.

Residual confounding cannot be excluded, as not all potential variables such as comorbidity burden, inflammatory markers, or detailed hydration practices were available.

Furthermore, the single-center nature of the study may limit the generalizability of the findings to other healthcare systems or Hospital-at-Home models.

Despite these limitations, the large sample size, real-world setting, and consistent assessment of functional and cognitive variables strengthen the validity of our findings.

## 6. Future Directions

Future research should focus on prospective studies aimed at identifying early predictors of hypernatremia in HaH and institutionalized populations. The development and evaluation of structured hydration monitoring protocols, combined with caregiver education, may help reduce the incidence of this complication. Additionally, integrating hypernatremia into frailty assessment frameworks could improve risk stratification and decision-making in advanced care planning. Multicenter studies are also warranted to confirm our findings and explore potential differences across healthcare systems and models of HaH delivery. Future studies should also explore the role of digital monitoring tools and artificial intelligence in early detection of dehydration risk in HaH settings.

## 7. Conclusions

Hypernatremia was significantly more prevalent among institutionalized patients receiving Hospital-at-Home care than among those treated in their own homes. In institutional settings, hypernatremia was closely associated with severe functional dependency, advanced cognitive impairment, and extremely high short- and medium-term mortality. These findings suggest that hypernatremia should be regarded as a marker of advanced frailty rather than an isolated electrolyte abnormality. Strengthening preventive strategies and hydration monitoring in HaH programs and long-term care facilities may help improve care quality and support timely, patient-centered decision-making in this vulnerable population. 

## Figures and Tables

**Table 1:** Baseline characteristics of Hospital-at-Home patients according to care setting.

Variable	HaH-Home (n = 2701)	HaH-Institution (n = 1800)	*p* Value
Age, years, mean ± SD	69.5 ± 18.0	90.1 ± 8.0	<0.001
Functional dependency (Barthel Index)	Lower	Higher	<0.001
Cognitive impairment (GDS)	Lower	Higher	<0.001
Hypernatremia prevalence, n (%)	22 (0.8)	57 (3.1)	<0.001

HaH: Hospital-at-Home; GDS: Global Deterioration Scale.

**Table 2:** Clinical characteristics and mortality according to severity of hypernatremia in institutionalized patients. The severity analysis included all patients with hypernatremia (n = 84), classified into mild, moderate, and extreme categories.

Variable	Mild (146–155)	Severe (156–165)	Extreme (>165)	*p* Value
	n = 35	n = 22	n = 27	
Age, years, mean	88	89	88	NS
Barthel Index, mean	12	13	10	NS
GDS stage, mean	6	6	6	NS
In-hospital mortality, %	37	27	33	NS
30-day mortality, %	74	41	63	0.041

Severity categories are based on serum sodium levels (mmol/L).

**Table 3:** Characteristics of institutionalized Hospital-at-Home patients according to hypernatremia status.

Variable	No Hypernatremia (n = 1743)	Hypernatremia (n = 84)	*p* Value
Age, years, mean ± SD	90 ± 8	88 ± 8	NS
Male sex, %	25	21	NS
Barthel Index, mean	15	11	0.041
GDS stage, mean	5.5	6.0	0.033

NS: not significant; GDS: Global Deterioration Scale.

**Table 4:** Comparison between in-hospital survivors and non-survivors among patients with hypernatremia.

Variable	Survivors (n = 56)	Non-Survivors (n = 28)	*p* Value
Age, years, mean	89	87	NS
Male sex, %	20	25	NS
Barthel Index, mean	12	11	NS
GDS stage, mean	5.9	5.5	NS
Serum sodium, mEq/L, mean	160	160	NS

NS: not significant; GDS: Global Deterioration Scale.

## Data Availability

The original contributions presented in this study are included in the article. Further inquiries can be directed to the corresponding author.

## Abbreviations

The following abbreviations are used in this manuscript:

HaH	Hospital-at-Home
ED	Emergency Department
GDS	Global Deterioration Scale
CI	Confidence Interval
